# Ponatinib as a Prophylactic or Pre-Emptive Strategy to Prevent Cytological Relapse after Allogeneic Stem Cell Transplantation in Patients with Philadelphia Chromosome-Positive Acute Lymphoblastic Leukemia Transplanted in Complete Cytological Remission †

**DOI:** 10.3390/cancers16112108

**Published:** 2024-05-31

**Authors:** Anna Candoni, Patrizia Chiusolo, Davide Lazzarotto, Chiara Sartor, Michelina Dargenio, Sabina Chiaretti, Cristina Skert, Fabio Giglio, Silvia Trappolini, Nicola Stefano Fracchiolla, Sara Medici, Paola Bresciani, Angela Cuoghi, Cristina Papayannidis

**Affiliations:** 1 Section of Haematology, Department of Medical and Surgical Sciences, University of Modena and Reggio Emilia, 41123 Modena, Italy; 2 Division of Hematology and Stem Cell Transplantation, ASUFC, 33100 Udine, Italy; 3 Hematology and Stem Cell Transplantation Unit, Fondazione Policlinico Universitario Agostino Gemelli IRCCS, 00136 Rome, Italy; 4 IRCCS Azienda Ospedaliero—Universitaria di Bologna, Istituto di Ematologia Seragnoli, 40126 Bologna, Italy; 5 Unità Operativa di Ematologia e Trapianto, Ospedale Vito Fazzi, 73100 Lecce, Italy; 6 Department of Translational and Precision Medicine, Sapienza University, 00161 Rome, Italy; 7 Hematology Unit, Ospedale Dell’Angelo, Mestre, 30174 Venice, Italy; 8 Hematology and Bone Marrow Transplantation Unit, San Raffaele Scientific Institute, 20132 Milan, Italy; 9 Hematology Department, University of Ancona, Azienda Ospedaliera Universitaria Ospedali Riuniti di Ancona, 60126 Ancona, Italy; 10 Fondazione IRCCS Ca’ Granda Ospedale Maggiore Policlinico, University of Milan, 20122 Milan, Italy

**Keywords:** acute lymphoblastic leukemia, ponatinib, TKI, allogeneic stem cell transplantation

## Abstract

**Simple Summary:**

The use of pre-emptive and prophylactic tyrosine kinase inhibitors (TKIs) after allogeneic hematopoietic stem cell transplantation (Allo-SCT) remains highly heterogeneous and very little is known about the use of third generation TKIs in this context. In this paper, we analyze the feasibility of maintenance with ponatinib administered after Allo-SCT to prevent cytologic relapse in a population of 48 patients with Philadelphia-positive acute lymphoblastic leukemia undergoing transplant while in complete cytologic remission. Although with the caution of the retrospective data, our analysis supports the feasibility of a ponatinib maintenance strategy after Allo-SCT, resulting in a low rate of discontinuation due to toxicity and a high probability of survival and relapse-free survival, particularly in the prophylactic group. In the majority of cases where a daily dose of 45 mg was started a dose reduction to 30–15 mg/day was required, which may be the appropriate dose to balance efficacy and tolerability.

**Abstract:**

The administration of TKIs after Allo-SCT in Philadelphia chromosome-positive acute lymphoblastic leukemia (Ph + ALL) remains controversial, and the TKI approach (prophylactic, pre-emptive or salvage) is still heterogeneous in transplant centers. In this context, very little is known about the feasibility and safety of third-generation TKIs. In this paper, we analyze the efficacy and safety of ponatinib (PONA) administered after Allo-SCT to prevent cytologic relapse of Ph + ALL. This is a multicenter observational study including 48 patients (pts) with Ph + ALL (median age 49 years) who received PONA after Allo-SCT while in complete cytological remission (cCR); 26 (54%) had positive minimal residual disease (MRD pos) before Allo-SCT. PONA was administered after Allo-SCT prophylactically (starting with MRD neg) in 26 pts or pre-emptively (starting with MRD pos post-SCT and without hematological relapse) in 22 pts. Patients treated prophylactically with PONA started treatment earlier, at a median of 4.3 months (range 1.5–6) after Allo-SCT, than those treated pre-emptively, who started PONA at a median of 7.4 months (range 2–63) after Allo-SCT (*p* = 0.01). The median starting dose of PONA was 30 mg/day (range 15–45). A dose reduction was required in 10/48 (21%) of cases, but a permanent discontinuation of PONA, due to toxicity, was required in only 5/48 pts (10.5%). No deaths due to PONA-related adverse events (AEs) were reported. The median follow-up time after Allo-SCT was 34 months (range 7.7–118). At the last follow-up, the median duration of PONA therapy was 22 months (range 2–100). The 5-year OS and RFS after Allo-SCT were 92% and 71%, respectively. The 5-year RFS after Allo-SCT of pts who received PONA prophylaxis was 95%, and it was 57% for those who received PONA pre-emptively (log-rank *p* = 0.02). In conclusion, this multicenter analysis of 48 patients with Ph + ALL undergoing Allo-SCT while in CcR, although with the caution of the retrospective data, supports the feasibility of PONA maintenance strategy after Allo-SCT with a low rate of discontinuations (10.5%) due to PONA-related AE.

## 1. Introduction

Philadelphia-positive acute lymphoblastic leukemia (Ph + ALL) still has a poor prognosis, and allogeneic hematopoietic stem cell transplantation (Allo-SCT) remains an important therapeutic option, although many advances have been made in biological knowledge and therapeutic approaches of this disease [1,2,3,4,5]. The main concerns after Allo-SCT are still transplant-related mortality and leukemia relapse prevention [1,2,3,6,7].

The use of tyrosine kinase inhibitors (TKIs) in the induction and consolidation phase of Ph + ALL therapy has improved clinical outcomes and is now considered a key component of the therapeutic program [1,2,3,4,5]. However, the appropriate administration of TKIs following Allo-SCT in Ph + ALL remains controversial, and the TKI approach (prophylactic, pre-emptive or salvage) is still heterogeneous across transplant centers, although the European Bone Marrow Transplantation Society (EBMT) published specific related recommendations in 2016 [6,7,8]. In this context, little is known about the efficacy and safety of third-generation TKIs after Allo-SCT [6,9].

Currently, ponatinib (PONA) is the only third-generation TKI available for the treatment of patients with Ph + ALL. It is a potent inhibitor of both the native BCR-ABL tyrosine kinase and isoforms carrying mutations responsible for resistance to other targeted drugs (including imatinib, dasatinib and nilotinib). PONA is also able to target several other kinases and is therefore classified as a multi-targeted kinase inhibitor [1,10,11,12].

Here, we report the results of a multicenter study to analyze the feasibility and safety profile of PONA maintenance therapy administered after Allo-SCT to prevent the Ph + ALL cytologic relapse.

## 2. Materials and Methods

This is a multicenter observational retrospective study that was conducted in accordance with the Declaration of Helsinki and was reviewed and approved by the Ethics Committee of FVG. The primary endpoint of this study is to evaluate feasibility and safety of PONA maintenance therapy after Allo-SCT. A total of 10 Italian hematology centers were involved. All patients provided informed consent for the collection of clinical data.

The study’s inclusion criteria consisted of all of the following: (a) age 18 years or older, (b) Ph + ALL undergoing Allo-SCT, (c) cytologic remission at the time of Allo-SCT, (d) administration of PONA for prophylactic or pre-emptive purpose (no salvage), (e) no others TKI post-Allo-SCT and (f) Allo-SCT for Ph + ALL between 2016 and 2022, regardless of conditioning regimen, donor type and graft source.

Post-transplant TKI treatment was defined as prophylactic if started in the presence of cytologic remission with negative minimal residual disease (MRD neg), and it was defined as pre-emptive if started in the presence cytologic remission but with a positive MRD (MRD pos) [8].

Complete cytological remission (CR) was defined as the presence of bone marrow lymphoid blasts below 5% in the absence of circulating blasts and platelets >100 × 10^9^/L. Cytologic relapse (REL) was defined as the recurrence of bone marrow blasts >5% or extramedullary leukemia in patients previously in CR. Pre-transplant MRD was assessed within 30 days prior to Allo-SCT reinfusion by quantitative PCR (qPCR) to detect BCR-ABL transcripts. MRD negativity (pre- and post-Allo-SCT) was defined by the absence of detectable BCR-ABL mRNA transcripts by real-time qPCR.

The timing of Allo-SCT, conditioning regimens administered, GVHD prophylaxis, and timing of MRD monitoring (BCR-ABL) after Allo-SCT were evaluated by each institution according to transplant protocols.

Adverse events were defined and graded according to the National Cancer Institute Common Terminology Criteria for Adverse Events, version 3.0 or 4.0.

### Statistical Analysis

Continuous variables were analyzed using descriptive statistics (arithmetic mean, standard deviation, median, minimum, and maximum). Categorical variables were compared using the chi-square test (two-sided test); a *p*-value < 0.05 was considered statistically significant. The survival curves of the study population were constructed using the Kaplan–Meier method and compared by log-rank test.

Overall survival (OS) of the entire population was measured from the time of Allo-SCT until death from any cause. Disease-free survival (DFS) of the study population was defined as the time after Allo-SCT between the start of TKI and the cytologic relapse (REL) or death. Patients who did not relapse were censored at the date of death or last follow-up.

The data were analyzed using the software MedCalc, version 12.5.0.0 (MedCalc Software bvba, Ostend, Belgium).

## 3. Results

### 3.1. Patient Characteristics

A total of 48 patients (pts) with Ph + ALL (median age 49 years; range 20–70) who received PONA after their Allo-SCT) were included (Table 1). Before transplant, 28 patients received dasatinib, 12 imatinib and 4 PONA as a part of frontline therapy (±chemotherapy). The donors were 24 matched unrelated donors (MUD), 11 HLA identical siblings, 9 haploidentical donors and 4 cord blood. All 48 pts received Allo-SCT while in complete cytological remission (cCR), and 26 pts (54%) had positive minimal residual disease (MRD pos) before Allo-SCT. BCR-ABL1 transcripts, conditioning regimens and donor source are detailed in Table 1. PONA was administered after Allo-SCT prophylactically (starting with MRD neg post-SCT) in 26 pts (54%) or pre-emptively (starting with MRD positivity post-SCT but without hematological relapse) in 22 pts (46%).

### 3.2. Ponatinib Treatment and Toxicity

The median time between Allo-SCT and PONA start was 4.8 months (range, 1.5–63 months). The 26 pts treated prophylactically (with MRD neg) with PONA started treatment earlier, at a median of 4.3 months (range 1.5–6) after Allo-SCT, than the 22 pts treated pre-emptively (with MRD pos), who started PONA at a median of 7.4 months (range, 2–63) after Allo-SCT (*p* = 0.01). The median starting dose of PONA was 30 mg/day (range, 15–45). In addition to PONA, 10 pts received donor lymphocyte infusions (DLIs). All DLI-receiving patients were included in the pre-emptive group (Table 2). The median duration of PONA treatment was 22 months (range, 2–100).

The main differences between prophylactic and pre-emptive PONA maintenance strategy are reported in Table 2. A significantly higher number of patients in the prophylactic group started with a lower dose of PONA (15 mg; 86% vs. 36%, *p* = 0.002), while a significantly higher number in the pre-emptive group started with a full dose of PONA (45 mg; 36% vs. 4%, *p* = 0.07).

**Figure 1:**
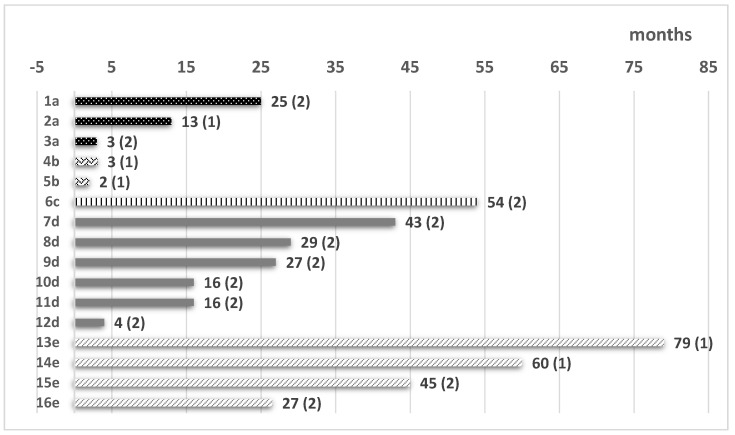
Patients (pts) who discontinued PONA therapy after Allo-SCT. Pts 1a–3a, artery disease. Pts 4b–5b, intolerance. Pt 6c, second Allo-SCT. Pts 7d–12d, leukemia relapse. Pts 13e–16e, stop PONA for long-lasting cytologic remission with MRD negativity. Legend: (1) PONA prophylaxis (pts MRD neg); (2) PONA pre-emptive (pts MRD pos).

As reported in Table 2, a reduction in the initial dose was required in 10/48 (21%) of cases (mainly in those receiving an initial dose of 45 mg/day), and a transient discontinuation is required in 5/48 (10.5%) cases. A permanent discontinuation of PONA, due to related adverse events (AEs), was required in 5/48 pts (12% in the prophylactic and 10% in the pre-emptive group, *p* = ns). In addition, six patients stopped PONA due to ALL cytologic relapse (6/6 in the pre-emptive group), four stopped the drug for a long-lasting MRD negativity (as per medical decision) and one underwent a second Allo-SCT procedure for a persistence of MRD positivity-Figure 1.

Detailed data on the safety profile of PONA are reported in Table 3. No deaths due to PONA-related adverse events were reported. Grades II-III hepatotoxicity occurred only in the pre-emptive group (4/22–20% of cases) and cardiovascular events (of any grade) occurred in only 1/26 pts (4%) in the prophylactic group (1 lower-limb peripheral artery disease) and in 4/22 (20%) in the pre-emptive group (2 peripheral artery stenosis, 1 hypertension and 1 atrial fibrillation). Other documented AEs, without significant differences in the two groups, were pancreatitis (grades II–III) in three cases (6%); hematologic toxicity (grades II–IV) in three cases (6%); fluid retention (grade I) in two cases (4%); and nausea (grade II), myalgia (grade II) and rash (grade II) each in one case, respectively.

### 3.3. Survival Analysis

**Figure 2:**
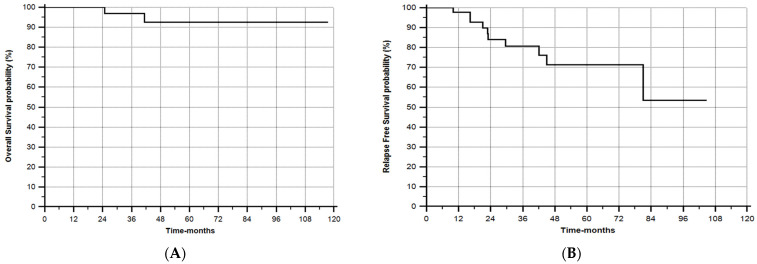

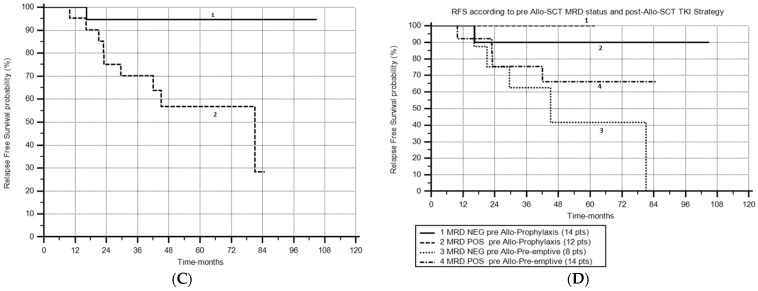
(**A**) Overall survival after Allo-SCT (92% at 60 months) (0 deaths in prophylaxis group and 2 deaths in the pre-emptive group). (**B**) Relapse-free survival (RFS) from the start of PONA—all 48 cases (71% at 60 months). (**C**) RFS according to the strategy (1 prophylaxis vs. 2 pre-emptive = dot line). Median RFS = NR in prophylaxis group and 81 months in pre-emptive group; log-rank, *p* = 0.02. (**D**) RFS according to the pre-Allo-SCT MRD status (pos or neg) and to the post-Allo-SCT PONA strategy (prophylaxis or pre-emptive). For RFS evaluation, the event was death or morphologic relapse. Only in the pre-emptive group, DLI + PONA in 10/22 cases (45%).

The mean and median follow-up times after Allo-SCT were 40 ± 26 and 34 months (range 7.7–118), respectively. The cytologic relapse rate under PONA maintenance therapy was 17% (8/48), with seven events in the pre-emptive group and only one event in the prophylactic group (*p* = 0.017). The 5-year probability of OS and RFS after Allo-SCT was 92% and 71%, respectively (Figure 2A,B). The 5-year RFS after Allo-SCT of pts who received PONA as prophylaxis was 95%, and it was 57% for those who received PONA pre-emptively (log-rank *p* = 0.02) unless that the pre-emptive group received, in addition to PONA, the DLI in 45% of cases (10/22) (Figure 2C). As reported, the median RFS was not reached in the prophylactic group, and it was 81 months in the pre-emptive group (Figure 2C). The RFS, according to the pre-Allo-SCT MRD status (pos or neg) and to the post-Allo-SCT PONA strategy (prophylaxis or pre-emptive), was reported in Figure 2D.

## 4. Discussion

The relapse rate after Allo-SCT is still a concern for Ph + ALL patients, ranging from 30 to 40%, according to the recent registry studies [13,14,15]. The results of Allo-SCT in Ph + ALL in Italy were provided by the GITMO Ph + ALL study (441 adult patients with Ph + ALL transplanted between 2005 and 2017), which reported a 5-year relapse rate of 32%. [13]. The data from this study, in terms of disease-free survival (DFS) and transplant related mortality (TRM), were in line with other recently published data from the European EBMT registry and the Japanese Society for Hematopoietic Stem Cell Transplantation-JSHCT registry [13,14,15].

Several salvage options are now available to treat cytologic relapse following Allo-SCT, including drug-conjugated monoclonal antibodies (inotuzumab), bispecific antibodies (blinatumomab), and CAR-T therapy [1,2,4,5,7]. However, the molecular target therapy with TKIs remains a significant preventive option for cytological relapse post-Allo-SCT, and the EBMT provided specific recommendations on this topic in 2016 [8,9]. Despite the EBMT indications, the use of pre-emptive and prophylactic TKIs after transplant remains limited and highly heterogeneous; also, in the GITMO Ph + ALL study cohort, only 30% of patient received a maintenance therapy, with TKIs following Allo-SCT [16,17,18,19,20,21].

Major limitations to TKI use after Allo-SCT include a lack of prospective randomized trials, concerns regarding tolerability, optimal dose, duration of maintenance therapy and best strategy to adopt (prophylaxis or pre-emptive) [6,16,17]. It is well known that the different TKIs have distinct safety profiles, and the major extra-hematologic toxicities for first- and second-generation TKI (imatinib, dasatinib, and nilotinib) were infections, pleural effusions (dasatinib), gastrointestinal toxicity, cutaneous rash and edema. For PONA (third-generation TKI), the main concern is the cardiovascular risks [1,6,7,9].

Data on post-transplant maintenance with third-generation TKIs (such as PONA) are very scarce [18,22,23]. In addition, the few studies that have been published so far included both patients transplanted with active cytologic disease and cases that were treated with PONA after Allo-SCT for a cytologic relapse [18,22].

Our present analysis was based on a cohort of patients with Ph + ALL, all of whom were transplanted in cytologic remission (and 46% were also in molecular remission). All patients had received only PONA in the post-Allo-SCT phase (no other TKIs), and the therapeutic strategy was exclusively prophylactic (in 26/48 cases) or pre-emptive (in 22/48 cases); no cases were treated for cytologic relapse. The characteristics of our population receiving PONA (48/48 in complete cytological remission both at the time of Allo-SCT and at the PONA start), while acknowledging the limitations of an observational analysis, allow us to highlight some aspects that are worth reporting and that we believe may be useful in clinical practice. Specifically, as reported in Table 2, we found that patients treated with prophylaxis received a significantly lower dose of PONA (median 15 mg vs. 30 mg, *p* = 0.038) and experienced fewer significant adverse events (Table 3). Particularly, we underline that the cardiovascular events of any grade were rare, especially in the prophylactic group (only one event), and none of them was fatal (Table 3). Another crucial point regarding TKIs post-Allo-SCT is their optimal doses, given that, in a transplanted population, the ordinary doses could be not well tolerated. In the present experience, a PONA dose reduction was required in only 8% of patients in prophylactic group, compared to 36% of patients that required a dose reduction in the pre-emptive group (*p* = 0.02)-Table 2. Furthermore, four patients who achieved a long-lasting MRD negativity (two in the PONA prophylactic group and two in the PONA pre-emptive group) discontinued PONA therapy after 79, 60, 45 and 47 months, respectively, based on physicians’ decision (Figure 1). All of these patients, after a median of 15 months from PONA interruption, were MRD-negative [24].

As reported in Figure 2B,C, the RFS of the entire population treated with PONA was very good (71% at 60 months), with a significantly better RFS in the prophylactic group (95% vs. 57%, *p* = 0.02), despite the fact that, in the pre-emptive group, in addition to PONA, DLI was administered in 10/22 (45%) of patients (while no cases received DLI in the prophylaxis group) [25].

Therefore, in the cohort of patients we analyzed, the largest group reported to date who received maintenance with PONA after Allo-SCT, we confirm the feasibility with a favorable safety profile of PONA treatment, particularly in the prophylactic group (MRD neg), for which lower PONA doses were used [11,23]. What we observed confirms the favorable data already reported on post-Allo-SCT maintenance therapy with first- and second-generation TKIs and is in line with what is indicated by both the EBMT-2016 and Leukemia NET-2024 recommendations, which support the post-transplant maintenance with TKIs in Ph + ALL as both a prophylactic and pre-emptive strategy [1,2,6,8]. Particularly, we think that TKI prophylaxis after Allo-SCT will play a significant role in relapse prevention in those patients with high-risk characteristics before transplant (e.g., IKAROS plus or patients transplanted in CR > 1) regardless of the MRD status at Allo-SCT [6]. Given that, with newer and effective induction programs and closer monitoring of MRD, a subset of Ph + ALL patients might not be further eligible for transplantation, we expect that, in the future, Allo-SCT will be reserved for patients with one or more poor risk characteristics for whom post-Allo-SCT maintenance therapy would be highly recommended.

## 5. Conclusions

In conclusion, data obtained from this analysis, in a population of Ph + ALL undergoing Allo-SCT while in CcR, although with the caution of the retrospective data, support the feasibility of PONA as a maintenance strategy after Allo-SCT, resulting in a low rate of discontinuation due to PONA-related toxicity with a high probability of OS and DFS. However, in the majority of cases where a daily dose of 45 mg was started a dose reduction to 30–15 mg/day was required, which could be the appropriate dose to balance efficacy and tolerability [26].

## Figures and Tables

**Table 1:** Ph + ALL and transplant characteristics (all 48 cases).

Age, Median Years (Range)	49	(20–70)
** BCR-ABL1 transcript type**	**No.**	**%**
p190	29/48	60%
p210	16/48	33%
p190/p210	3/48	7%
** Leukemia status at Allo-SCT**		
cCR	48/48	100%
MRD positive	26/48	54%
MRD negative	22/48	46%
** Conditioning regimen**		
MAC	40/48	83%
RIC	8/48	17%
** Donor type**		
MUD	24/48	50%
HLA-id sibling	11/48	23%
HAPLO	9/48	19%
CB	4/48	8%
** PONA maintenance post-Allo-SCT**		
Prophylactic strategy (MRD neg)	26/48	54%
Pre-emptive strategy (MRD pos)	22/48	46%

Notes: cCR, cytologic Complete Remission; MRD, minimal residual disease; MUD, HLA-matched unrelated donor; HLA-id, HLA-matched sibling donor; HAPLO, HLA-haploidentical donor; CB, cord blood; PONA, ponatinib.

**Table 2:** **2A**-ponatinib (PONA) therapy according to strategy (prophylactic vs. pre-emptive); **2B**-PONA dose; **2C**-PONA dose modulation.

2A-PONA Therapy	Total Cases	Prophylactic Therapy (26)	Pre-Emptive Therapy (22)	*p*-Value
**Cytologic Remission at Allo-SCT**	48/48 (100%)	26/26 (100%)	22/22 (100%)	
**MRD positive at Allo-SCT**	26/48 (54%)	12/26 (46%)	14/22 (64%)	
**MRD negative at Allo-SCT**	22/48 (46%)	14/26 (54%)	8/22 (36%)	
**Time from Allo-SCT to PONA start,** **months median (range)**	4.8 (1.5–63)	4.3 (1.5–6)	7.4 (2–63)	0.01
**Duration of PONA therapy,** **months’ median (range)**	22 (2–100)	15 (2–100)	29 (6–80)	
**2B-PONA DOSE**				***p*-Value**
**Starting dose (mg/day)** **median (range)**	30 (15–45)	15 (15–45)	30(15–45)	0.038
PONA 15 mg/day	29/48 (60%)	21/26 (86%)	8/22 (36%)	0.002
PONA 30 mg/day	10/48 (21%)	4/26 (15%)	6/22 (28%)	ns
PONA 45 mg/day	9/48 (19%)	1/26 (4%)	8/22 (36%)	0.007
**PONA + DLI**	10/48 (21%)	0/26	10/22 (45%)	0.00009
**2C-PONA DOSE MODULATION**				***p*-Value**
**Dose increase during therapy**	7/48 (15%)	2/26 (8%)	5/22 (23%)	ns
**Dose reduction during therapy**	10/48 (21%)	2/26 (8%)	8/22 (36%)	0.02
**Transient discontinuation**	5/48 (10.5%)	4/26 (16%)	1/22 (5%)	ns
**Permanent discontinuation**				
**All causes**	16/48 (33%)	5/26 (19%)	11/22 (50%)	0.03
**Only AE**	5/48 (10.5%)	3/26 (12%) ^a^	2/22 (10%) ^b^	ns

Notes: ^a^ 3 cases of intolerance (myalgia, rash and fluid retention) and 1 lower 0 limb peripheral artery disease (PONA 15 mg); ^b^ 1 femoral artery stenosis (PONA 45 mg) and 1 subclavian artery stenosis (PONA 30 mg).

**Table 3:** Toxicity during PONA therapy.

	All	Prophylaxis (26 pts)	Pre-Emptive (22 pts)
Hepatic toxicity (grade II-III)	4/48 (8%)	/	4/22 (20%)
Pancreatic toxicity (grade II-III)	3/48 (6%)	2/26 (8%)	1/22 (5%)
Hematologic toxicity (grade II-IV)	3/48 (6%)	1/26 (4%)	2/22 (10%)
Nausea (grade II)	1/48 (2%)	1/26 (4%)	/
Fluid retention (grade I)	2/48 (4%)	1/26 (4%)	1/22 (5%)
Myalgia (grade II)	1/48 (2%)	1/26 (4%)	/
Rash (grade II)	1/48 (2%)	/	1/22 (5%)
Cardiovascular events (any grade)	5/48 (10%)	1/26 (4%) ^a^	4/22 (20%) ^b^

Notes: ^a^ lower-limb peripheral artery disease (PONA 15 mg); ^b^ 1 femoral artery stenosis (PONA 45 mg), 1 subclavian artery stenosis (PONA 30 mg), 1 hypertension and 1 transient atrial fibrillation.

## Data Availability

The authors will make the raw data supporting this article’s conclusions available upon request.
